# Coelomic Fluid of *Eisenia fetida* Ameliorates Cetuximab to Reduce K-Ras and Vimentin Expression through Promoting RUNX3 in an AOM/DSS-Induced Colitis Associated Colon Cancer

**DOI:** 10.1155/2020/9418520

**Published:** 2020-07-19

**Authors:** Sofy Permana, Reyudzky Putri Fityanti, Eviana Norahmawati, Agustin Iskandar, Erika Desy Anggraini Mulyadi, Agustina Tri Endharti

**Affiliations:** ^1^Department of Biology, Faculty of Mathematics and Natural Sciences, Universitas Brawijaya, Malang, Indonesia; ^2^Master Program in Biomedical Sciences, Faculty of Medicine, Universitas Brawijaya, Malang, Indonesia; ^3^Department of Pathology Anatomy, Faculty of Medicine, Universitas Brawijaya, Malang, Indonesia; ^4^Department of Parasitology, Faculty of Medicine, Universitas Brawijaya, Malang, Indonesia; ^5^Undergraduate Program of Biology, Faculty of Mathematics and Natural Sciences, Universitas Brawijaya, Malang, Indonesia; ^6^Biomedical Central Laboratory, Faculty of Medicine, Universitas Brawijaya, Malang, Indonesia

## Abstract

Ulcerative colitis is a major risk factor that increases the occurrence of colorectal cancer. In colorectal cancer due to colitis, intestinal inflammation plays an important role which causes DNA damage. The aim of this study is to investigate the anticancer effect of coelomic fluid of *Eisenia fetida* (CFEF) and *cetuximab* combinations. Colitis associated colon cancer was induced in BALB/c mice by DSS/AOM. The mice were randomly divided into six groups: group 1 received vehicle (control), groups 2–6 received DSS/AOM, groups 3–5 received cetuximab + CFEF (30, 60, or 120 mg/kgBW), and group 6 received CFEF only. After the 12^th^ week of treatments, the colon tissues were removed for histological examination and immune-fluorescence. Intestinal Epithelial Cells (CECs) were analyzed by flow cytometer. Administration of CFEF significantly decreased the severity of DSS/AOM-induced CAC in a dose-dependent manner. The combinations of CFEF-cetuximab were revealed by histological change. The CFEF significantly reduced the severity scores (*P* < 0.05). The combinations of CFEF-cetuximab significantly inhibited K-Ras and vimentin expressions, whereas the percentage of RUNX3 significantly increased in CECs. The increasing of RUNX3 could prevent EMT, so that it can decrease K-Ras and vimentin to suppressed cell invasion and migration by CFEF. Our results suggest that CFEF has the therapeutic potential to CAC.

## 1. Introduction

Colorectal cancer is the most common malignant tumor with high prevalence and low 5-year survival [1, 2]. Ulcerative colitis is a major risk factor that increases the occurrence of colorectal cancer. In colorectal cancer due to colitis, intestinal inflammation plays an important role which causes DNA damage, increased cell proliferation, decreased tumor suppressors, and also apoptosis [3–5]. These things occur through signal transduction from Epidermal Growth Factor Receptor (EGFR) bound with ligands to the nucleus via MAPK pathway. In the MAPK pathway, K-Ras plays a role in regulating cell proliferation and cell survival. Thus, K-Ras is considered to have a significant potential therapeutic value [3–5]. In addition, tumor budding is also included in colorectal cancer [4, 5]. Tumor budding is a part of epithelial mesenchymal transition (EMT). In the EMT process, there is an upregulation of vimentin. Impaired EMT activation has been known to play a role in cancer metastases [5, 6].

The migration, as one of the factors involved in cancer metastasis, is a process known as EMT. The EMT phenotype is characterized by the loss of cell-to-cell adhesion and remodeling of the epithelial molecule E-cadherin and mesenchyme markers such as vimentin. EMT has been shown to prevent overexpression of Runt-Related Transcription Factor-3 (RUNX3), which facilitates metastasis, cell invasion migration, and loss of RUNX3 in epithelial cells [7–9]. Runt-Related Transcription Factor-3 is a transcription factor known for its tumor suppressor activity and more recently has been implicated in cancer metastasis. RUNX3 is an interpretation factor known for its tumor suppressor activity and lately has been involved in malignant growth metastasis. The Runt-Related Transcription Factor-3 (RUNX3), which plays a significant part in cell proliferation [8–13], has been shown to play a tumor suppressor role in several types of cancers and its expression levels are downregulated in cancer. It is currently clear that EMT has implications on cancer metastasis by triggering the loss of cell-cell adhesion to facilitate the invasion of cancer cell. However, the mechanism of RUNX3-mediated suppression of cancer metastasis remains unclear and the role of RUNX3 in colorectal cancer has not yet been well studied [11–14]. Further biochemical studies need to be followed up to investigate the mechanistic principles involved in RUNX3-mediated inhibition of EMT in colitis associated colon cancer. In the development of EMT, cancer cells lose the properties of epithelial cells and develop mesenchymal cell properties, including overexpression of vimentin [12–16]. The potential mechanisms of cetuximab combination mediated EMT are not well understood. Cetuximab is a target therapy for colorectal cancer that works in the EGFR extracellular domain by inhibition of K-Ras [11, 14].

Cetuximab is a target therapy for colorectal cancer that works in the EGFR extracellular domain by inhibiting MAPK pathway. Although colorectal cancer patients with metastasis have been treated with cetuximab, unfortunately, cetuximab can cause many side-effects such as diarrhea, nausea, vomiting, and skin rashes [17–19].

According to Endharti et al. [8–10], the combination of coelomic fluid with 5-fluorouracil has anticancer potential for colorectal cancer. Coelomic fluid in earthworms is secreted through dorsal pores in their skin. This fluid have been shown to have antioxidant, antibacterial, anti-inflammatory, and antitumor activities [18, 19]. Some of the active compounds in coelomic fluid are lectins, lysenin, phenoloxidase, antibacterial peptides polysaccharides, fibrinolytic enzymes, and proteases (Endharti et al.) [8–10]. It is also established that coelomic fluid has a cytotoxic and antiproliferative effect on cancer activity that may increase apoptosis in HT-29 cells (Permana et al.) [9]. *Eisenia fetida* is a type of earthworm that is easily maintained and it can produce more coelomic fluid than other types of earthworms [16–19]. This coelomic fluid has also found several bioactive compounds, exhibiting a variety of biological functions [17–20].

Coelomic fluid is also known to have a cytotoxic and antiproliferative effect on cancer activity that can increase apoptosis in HT-29 cell lines [18–20]. The combination of coelomic fluid with 5-fluorouracil has anticancer potential for colorectal cancer [21–25]. *Eisenia fetida* is a species of earthworm that is easily maintained and it can produce more coelomic fluid than other species [22]. The use of coelomic fluid as a therapy only causes side-effects that can be ignored. Also because there have never been reports of the side-effects from therapies using coelomic fluid, coelomic fluid may be consumed continuously [24, 25].

Considering the interest in the possible anticancer properties of coelomic fluid, the main goal of this investigation was to evaluate the actions of coelomic fluid from *Eisenia fetida* on mice model colitis associated colorectal cancer. Nevertheless, to our knowledge, evidence testifying the antiproliferative property of CFEF in CAC is still limited. In this study, we demonstrated the antimetastasis effects and studied associated mechanisms of continuous CFEF intervention on an AOM/DSS mouse model.

## 2. Materials and Methods

### 2.1. Mice

Female BALB/c mice aged 8–10 weeks were housed in laboratory of animal, Faculty of Medicine, Universitas Brawijaya, were approved pathogen-free barrier facility at constant temperature, and were kept in controlled conditions of humidity (50 ± 10%), light (12-hour light/dark cycle), and temperature (25 ± 2°C). All the studies and animal protocols were performed in accordance with the institutional guidelines by The Institutional Animal Care and Use Committee (IAUCUC) by University of Brawijaya Ethical Committee (no. 1130-KEP-UB).

### 2.2. Coelomic Fluid Collection

Heat and cold shock methods were used to collect coelomic fluid (Endharti et al.) [8]. Briefly, the same quantities of *Eisenia fetida* (±20 g) were place in plastic box with wire mesh filter, rinsed with distilled water to remove adhering materials or particles, and then dried by using tissue paper. The gut was cleaned of organic matter after 48 hours as it was fed on filter paper. They were thoroughly washed with distilled water and then put in glass funnel, using hot water (40°C–50°C) in a glass beaker to give heat shock and earthworms were shocked by the use of ice cubes in a plastic box. Instead, the procedure was within three minutes to resolve the shock effect and give cold shock in a similar manner. Coelomic fluid *Eisenia fetida (*CFEF) was collected into tubes and stored in aliquots at −20°C.

### 2.3. The Precipitation of Coelomic Fluid

Coelomic fluid was precipitated according to Endharti et al. [8–10]. Briefly, coelomic fluid was precipitated by adding of 2 mM ammonium sulfate. The pellet collected by centrifugation was resuspended in 20 mM Tris-HCl (pH8.0) followed by centrifugation. Then, the pellet was resuspended in cold acetone by adding the acetone slowly, followed by 20-minute incubation. The remaining acetone was removed from the pellet by centrifugation. Protein concentrations were measured using NanoDrop spectrophotometer.

### 2.4. Induction of AOM/DSS-Induced Colitis Associated Colon Cancer (CAC)

Colitis associated colon cancer model was induced as previously described by Endharti et al. [8–10]. Briefly, mice were intraperitoneal injected with a single dose of 10 mg/kg azoxymethane (AOM; Sigma-Aldrich, USA) on day 1. Colon cancer was induced by cyclical DSS treatment, which consisted of 1 week of 3% DSS followed by 7 days of untreated water. One week after the AOM injection, mice were given four cycles of 3% dextran sulfate sodium (DSS) (Sigma-Aldrich, USA) and followed by one week of regular water. Mice housed under specific-pathogen-free conditions were divided into seven groups: group 1 (vehicle). Groups 2–7 were given AOM and 3% DSS. Groups 3–6 were treated with cetuximab (10 mg/kg BW). Groups 4–6 were also treated with CFEF at 30, 60, and 120 mg/g/BW, respectively. Group 7 was only given CFEF 120 mg/g/BW. Cetuximab and CFEF were injected intraperitoneally once a week during the four cycles of DSS treatment. One week after AOM injection, mice were given four cycles of DSS in their drinking water and then distilled water until the end of the experiment. Mice were sequentially killed randomly at the end of the 10^th^ week, and at least six mice were killed for each group at each time point. Colon tissues were collected for analysis by immunofluorescence and flow cytometry. The colitis development was detected using Fecal Occult Blood Test (FOBT).

### 2.5. Immunohistochemistry for IL-6 Expression

IL-6 expression in colon tissues was inspected using a standard immunohistochemistry method. Immunostaining was done on serial sections as described previously by Zeng et al. [11]. Briefly, the tissues were fixed in 10% formaldehyde and embedded in paraffin and then cut into 4 *μ*m sections. Tissue sections were, respectively, deparaffinized in xylene and rehydrated by grade alcohols. The tissue was incubated overnight at 4°C with primary antibody specific for IL-6 (Santa Cruz, CA, USA, dilution 1 : 200). The tissue sections were then incubated with biotin-labeled goat anti-mouse antibody (Sigma, USA) followed by exposure to avidin-peroxidase complex (Sigma, USA). Staining was developed with diaminobenzidine (DAB, Sigma) substrate and sections were counterstained with hematoxylin.

### 2.6. Immunofluorescence for K-Ras, Vimentin, E-Cadherin, and *β*-Catenin Expressions

Immunofluorescence was done on serial sections as described previously by Permana et al. [10]. Immunofluorescent analysis of colon tissue stained with antivimentin antibody (biolegend), anti-K-Ras antibody (MyBioSource), anti-E-cadherin antibody (Santa Cruz), and anti-β-catenin antibody (Santa Cruz). In brief, colon tissue was made into histopathology slide and deparaffinized. Then slides were incubated in chamber with buffer citrate for 5 minutes of 300 volts for antigen retrieval. Tissue sections were washed with 0.1% Triton-X 100 in PBS for 5 minutes and followed with incubation of 1% BSA for 30 minutes. After washing, tissue sections were incubated with the following each specific primary antibody for vimentin (BioLegend, USA, 1 : 200), K-Ras (MyBioSource, USA, 1 : 100), E-cadherin (Santa Cruz, Biotechnology., Inc. USA, 1 : 100), and *β*-catenin (Santa Cruz, Biotechnology. Inc. USA, 1 : 200), respectively, for 2 hours; each tissue section was followed by incubation with the secondary antibodies anti-mouse FITC (1 : 100) for 1 hour at room temperature. Slides were mounted with 4′,6-diamidino-2-phenylindole (DAPI, BioLegend, USA) (1 : 1000) for 5 minutes. Slides were observed under fluorescence microscope (OLYMPUS 1X71). The contrast and/or brightness adjustment was applied evenly over the whole field of the image. Image analysis was performed using FIJI/ImageJ-2 software (Bethesda, MD, USA).

### 2.7. Isolation of Colon Epithelial Cells (CECs)

Primary CECs were isolated using a protocol adapted from Endharti et al. [8–10]. Briefly, mice duodenal and colonic tissues were removed from mice flushed of luminal contents by removing the longitudinal muscle layer and washed using wash buffer (Hank's Balanced Salt Solution (HBSS), Mg^2+^ and Ca^2+^ free (Gibco), 100 U penicillin-streptomycin (Gibco), and 25 *μ*g ml^−1^ amphotericin B (Gibco). Intestinal tissues were cut into small pieces, suspended in 50 ml wash buffer, and inverted vigorously ten times, and the contents were allowed to settle for 1 minute. The supernatant was removed and a further four times washed off the settled contents. After the fifth wash, the settled contents were removed and suspended in wash buffer. Intestinal tissues were digested in 50 ml of a digestion buffer (75 U ml^−1^ collagenase type XI-0.5 mM DTT (Sigma-Aldrich), 4% FBS (Gibco)) in Dulbecco's Modification of Eagles Medium (DMEM) (Gibco). The tissue-containing digestion buffer was placed in an incubator of 37°C and allowed to shake for 3 hours at 200 rpm. The effluent was centrifuged at 200 ×*g*, for 5 minutes, at 4°C. The remaining pellet containing isolated CECs was suspended digestion buffer. This process was repeated four times. The resultant pooled supernatants were pelleted by centrifugation (1000 ×*g*) for 30 minutes at 4°C. The pellet of CECs were washed and resuspended in FACS buffer.

### 2.8. The Percentage of RUNX3 Using Flow Cytometry

The CECs (1 × 10^6^) cells were placed in 1.5 mL tube and resuspended in FACS buffer containing monoclonal antibodies anti-MMP2 conjugated with FITC (Bioscience) or anti-RUNX3 conjugated with PE (Bioscience) and incubated for 20 minutes at 4°C. After the staining, cells were washed twice and analyzed the following day on the FACS Calibur (BD Biosciences). Data were analyzed using Cell Quest-Pro software (Becton, Dickinson, USA).

### 2.9. Statistical Analysis

One-way ANOVA was used to analyze the data. The data outcome was defined as a mean ± standard deviation and the statistical significance of a distinction regarded significant with *P* < 0.05 between each group. The statistical assessment was carried out using SPSS software.

## 3. Results

### 3.1. CFEF Ameliorated DSS/AOM-Induced Colitis Associated Colon Cancer

**Figure 1:**
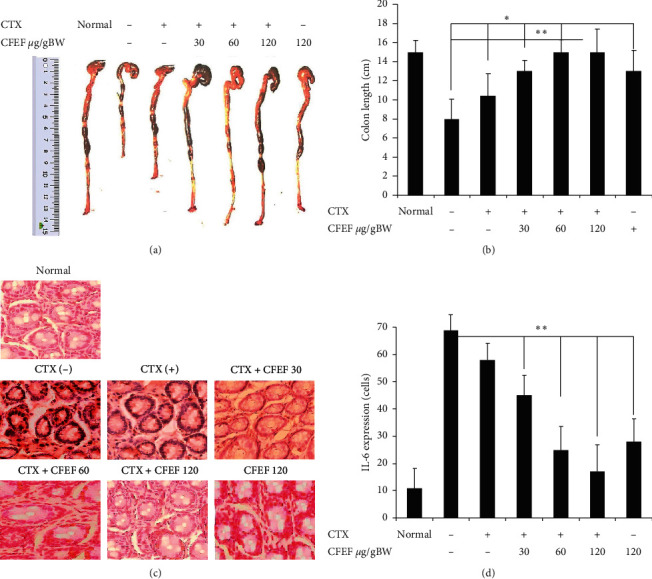
Coelomic fluid of *Eisenia fetida* (CFEF) ameliorated cetuximab AOM/DSS-induced colitis associated colon cancer. (a) We first analyzed severity of colitis to evaluate early impacts of CFEF. Colon length ratio in control and DSS/AOM-induced colitis associated colon cancer. (b) Mice were significantly protected against DSS/AOM-induced colitis associated colon cancer compared with control mice (*P* < 0.05). (c) Immunohistochemical staining of IL-6 antibody of colon tissue. (d) The number of IL-6 expression of the colons was significantly lower in CFEF-cetuximab-treated mice than control mice after AOM/DSS administration (*P* < 0.05). Results shown are mean + SD, with *n* = 6 replicates in each group. ^*∗*^*P* < 0.05, ^*∗∗*^*P* < 0.001.

We first analyzed severity of colitis to evaluate early impacts of CFEF. Colon length ratio in control and DSS/AOM-induced colitis associated colon cancer (Figure 1(a)). Mice were significantly protected against DSS/AOM-induced colitis associated colon cancer compared with control mice (*P* < 0.05) (Figure 1(b)). The expression of IL-6 showed that the AOM/DSS-treated mice with CFEF administration had significantly less inflammation compared to the AOM/DSS-treated mice (Figure 1(c)). CFEF-cetuximab-treated mice showed significant protection against 3% DSS/AOM-induced colitis associated colon cancer (*P* < 0.05) (Figure 1(d)). AOM/DSS-induced mice were significantly induced colon inflammation compared with control mice (*P* < 0.05). Data showed that the AOM/DSS-treated mice with CFEF administration had significantly less inflammation compared to the AOM/DSS-treated mice.

### 3.2. The Activity of the Enzyme Myeloperoxidase (MPO) Was Used to Evaluate Infiltration of Neutrophils

**Figure 2:**
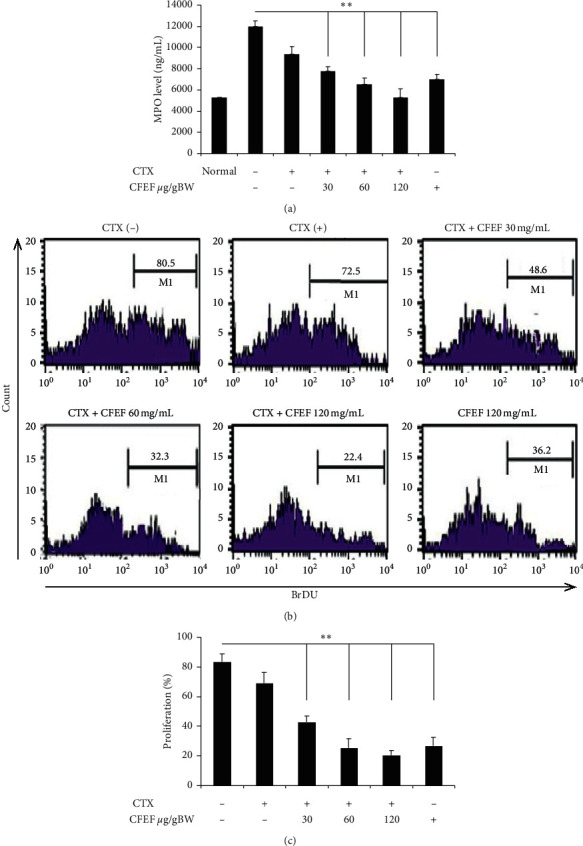
CFEF-cetuximab decreased MPO levels and proliferation in CECs cell. (a) MPO levels were measured from lysed colon cells. (b) Representative of CECs proliferation after therapies with cetuximab and various concentrations of CFEF (30, 60, and 120 mg/g BW) in each histogram were shown inside the panels. Percentages of CECs proliferation in each histogram are shown. (c) The percentage of CECs proliferation was showed by BrdU positive cells and analyzed using flow cytometry (FACS Calibur, Becton Dickinson). Results shown are mean + SD, with *n* = 6 replicates in each group. ^*∗*^*P* < 0.05, ^*∗∗*^*P* < 0.001.

MPO levels were measured from lysed colon cells (Figure 2(a)). The expression of cell proliferation was inhibited by CFEF-cetuximab in colon tissue. The expressions of proliferation were analyzed using flow cytometry. Representative result from each group inhibited proliferation in CFEF-cetuximab combination groups (Figure 2(b)). The percentage of BrdU positive cells indicated cell proliferation. The combination CFEF-cetuximab therapy groups have lower cell proliferation than cetuximab single therapy group (Figure 2(c)). Results shown were mean ± SD with *n* = 6 replicated in each group by ^*∗*^*P* < 0.05, ^*∗∗*^*P* < 0.001.

### 3.3. CFEF Plays a Major Role in Reducing Activated K-Ras Mediated Inflammation during Tumor genesis

**Figure 3:**
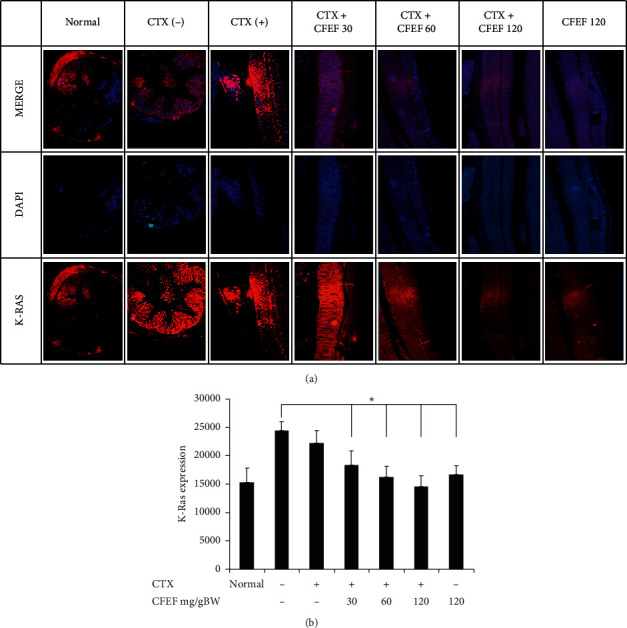
K-Ras expressions were reduced by CFEF-cetuximab in colon tissue. The expression of K-Ras after therapies with cetuximab and various concentrations of CFEF (30, 60, and 120 mg/g BW) were analyzed using immunofluorescence. The expression of K-Ras reduced in colon tissue by the decreasing of fluorescence intensity. (a) Representative images of K-Ras expression showed slightly lower on the combination therapy group. (b) The percentage of K-Ras expression decreased on combination therapy groups compared with cetuximab single group. Results shown are mean ± SD, with *n* = 6 replicates in each group. ^*∗*^*P* < 0.05, ^*∗∗*^*P* < 0.001.

To analyze the oncogene expression is involved in modulating responses to CFEF effects of inflammation. K-Ras expression was determined by using immune-fluorescence. As shown in Figure 3, the combination of cetuximab and CFEF exert an ameliorate anticancer activity on AOM/DSS-induced mice. The representative images shown in Figure 3 demonstrated a clear decreased K-Ras expression after treatment with CFEF alone or in combinations with CFEF and cetuximab. When the combination of CFEF and cetuximab was applied, the percentage of FAK expression was lower than cetuximab or CFEF alone (*P* < 0.05). Treatment with cetuximab only slightly reduced the expression of K-Ras (*P* < 0.05). These data indicated that K-Ras expression was decreased via CFEF and cetuximab combinations. CFEF at three doses significantly inhibited K-Ras expression compared to the control (Figure 3). These results indicate that CFEF can inhibit protein oncogene that was found to be particularly important in tumor progression.

### 3.4. Combination of Cetuximab and CFEF Restored Membrane Integrity by Inhibition of Vimentin Expression

**Figure 4:**
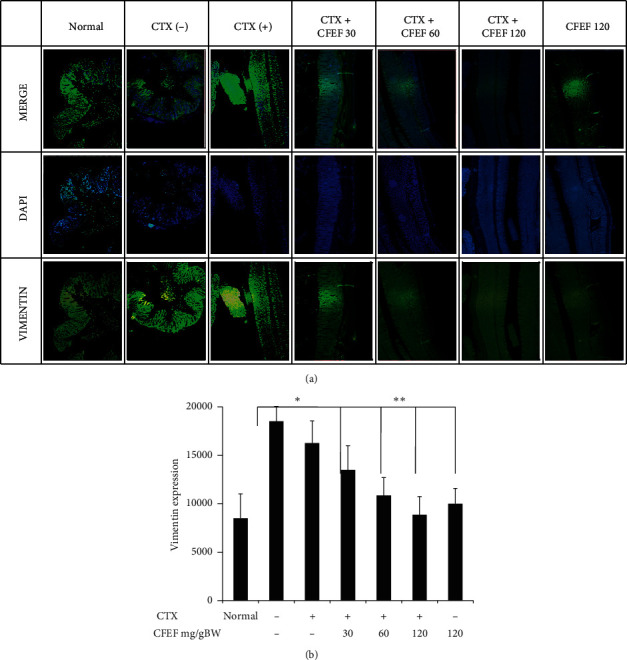
Vimentin expressions were reduced by CFEF-cetuximab in colon tissue. The expression of vimentin after therapies with cetuximab and various concentrations of CFEF (30, 60, and 120 mg/g BW) were analyzed using immunofluorescence. The expression of vimentin reduced in colon tissue by the decreasing of fluorescence intensity. (a) Representative images of vimentin expression showed slightly lower on the combination therapy group. (b) The percentage of vimentin expression decreased on combination therapy groups compared with cetuximab single group. Results shown are mean ± SD, with *n* = 6 replicates in each group. ^*∗*^*P* < 0.05, ^*∗∗*^*P* < 0.001.

To explore that CFEF have role in EMT process of cancer cell migration, invasion, and metastasis via vimentin, the colon tissue was observed by using vimentin staining performed by immune-fluorescence in colon tissues. In DSS-AOM only group, the expression of vimentin decreased compare with that of combination-treated group. Treatment with cetuximab only slightly reduced the expression of vimentin (*P* < 0.05). These data indicated that vimentin expression was decreased via CFEF and cetuximab combinations. CFEF at three doses significantly inhibited vimentin expression compared to the control (Figure 4).

### 3.5. Combination of Cetuximab and CFEF Induced by *β*-Catenin and E-Cadherin Expression

**Figure 5:**
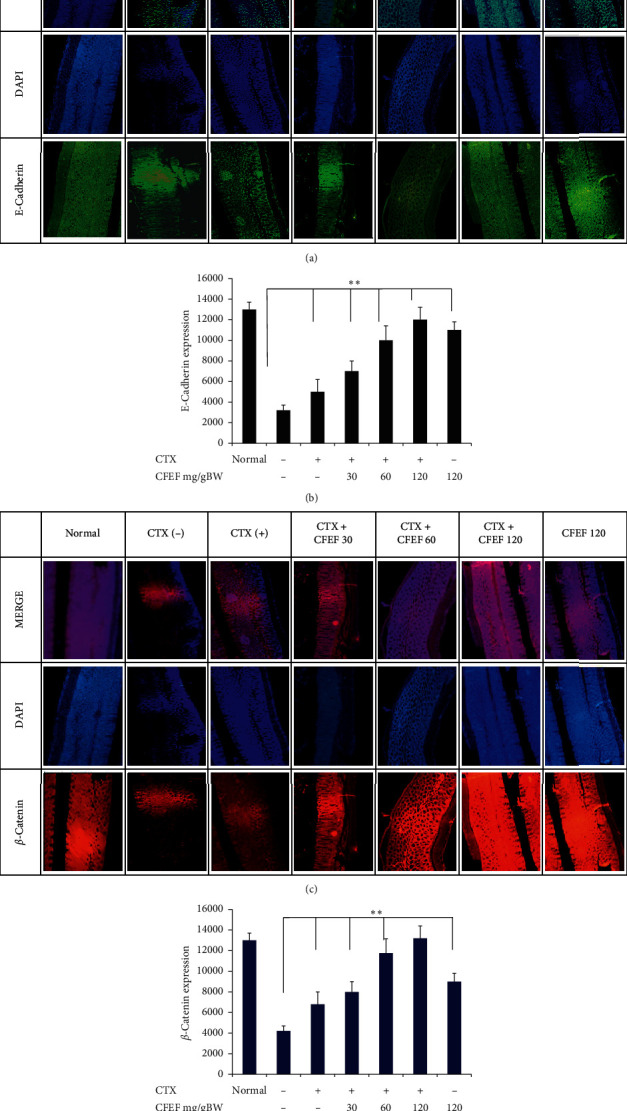
E-Cadherin and *β*-catenin expressions were reduced by CFEF-cetuximab in colon tissue. The expression of E-cadherin and *β*-catenin after therapies with cetuximab and various concentrations of CFEF (30, 60, and 120 mg/g BW) were analyzed using immunofluorescence. (a) Representative images of E-cadherin expression showed slightly higher on the combination therapy group. (b) The percentage of E-cadherin expression increased on the combination therapy groups compared with cetuximab single group. (c) Representative images of *β*-catenin expression showed higher on the combination therapy group. (d) The percentage of *β*-catenin expression increased on combination therapy groups compared with cetuximab single group. Results shown are mean ± SD, with *n* = 6 replicates in each group. ^*∗*^*P* < 0.05, ^*∗∗*^*P* < 0.001.

We further examined the effects of CFEF with or without combinations to the expression of *β*-catenin and E-cadherin. As shown in Figures 5(a)–5(d), CFEF enhanced the protein expression of E-cadherin. Treatment of combination of cetuximab and CFEF significantly increased the protein expression of *β*-catenin and E-cadherin. This study proved clear decreased *β*-catenin and E-cadherin expressions after treatment with CFEF alone or in combination with CFEF and cetuximab. When the combination of CFEF and cetuximab was applied, both expressions of *β*-catenin and E-cadherin were higher than cetuximab or CFEF alone (*P* < 0.05).

### 3.6. Combination of Cetuximab and CFEF Increases Percentage of RUNX3 in DSS/AOM-Induced Mice

RUNX3 is important proteins involved in cell cycle regulation [26]. Therefore, we wanted to determine if CFEF-cetuximab treatments influenced the abundance of RUNX3 in cancer cells. To do this, we analyzed cells that were expressed by RUNX3 by using flow cytometry.

To explore whether CFEF could regulate the improvement of tumor suppressor in CECs, the RUNX3 protein expression from DSS/AOM-induced mice was characterized by flow cytometry. The percentage of RUNX3 between control and treatment groups was significantly increased, *P* < 0.05, and for CFEF, *P* < 0.05 (Figure 5). In this study, there was a significant increase in the percentage of RUNX3. There is a clear direct relationship, indicating that an increase in the dosage of CFEF increased the percentage of protein.

### 3.7. RUNX3 is a Regulator of MMP-2 and MMP-9 for Invasiveness

**Figure 6:**
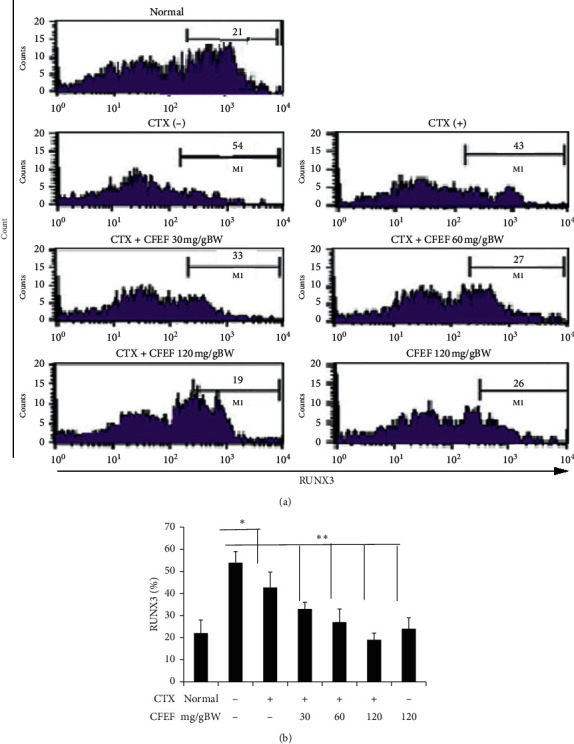
The percentage of RUNX3 was enhanced by CFEF-cetuximab in CECs cells. (a) Representative percentage of RUNX3 after therapies with cetuximab and various concentrations of CFEF (30, 60, and 120 mg/g BW) were analyzed using flow cytometry (FACS Calibur, Becton Dickinson) in each histogram as shown. Cells were gated from double positive cells (K-Ras^+^ and vimentin^+^ populations) and those populations that express RUNX3 were analyzed. (b) The percentage of RUNX3^+^ cell enhanced on combination therapy groups compared with cetuximab single group. Results shown are mean ± SD, with *n* = 6 replicates in each group. ^*∗*^*P* < 0.05, ^*∗∗*^*P* < 0.001.

To determine how RUNX3 inhibits cell migration and invasion, we focused on clarifying the relationship between RUNX3 and MMPs, which have been reported to participate in tumor progression. We found that RUNX3 overexpression significantly inhibited the expression of MMP-2 and MMP-9 in CECs (Figures 6(a) and 6(b)). As shown in Figure 6, the percentages of MMP-2 and MMP-9 were increased. Hence, we hypothesized that MMP-2 and MMP-9 were affected by the invasive effects. As shown in Figure 6, the cells treated with CFEF-cetuximab showed significantly lower invasiveness. Together, these data demonstrated that RUNX3 inhibits metastasis through MMP-2 and MMP-9. To investigate that RUNX3 has dependent mechanism of MMP2 and MMP9, we showed that RUNX2 regulates MMP2 and MMP9 expressions. We proved that percentage of both (RUNX3+ MMP-2+) and (RUNX3+ MMP-9+) cells significantly diminished in CECs on combination therapy groups (7(a)–7(d)). Together, these data demonstrated that RUNX3 inhibits metastasis through MMP-2 and MMP-9.

## 4. Discussion

Colorectal cancer is a malignant tumor that is the leading cause of death worldwide. These malignant tumors originate from the colon or rectum epithelium that penetrates the mucosal muscular layer. In colorectal cancer due to ulcerative colitis, DNA damage occurs that leads to increased cell proliferation, inhibition of tumor suppressors, and inhibition of apoptosis [7–10]. AOM can cause mutagenesis, so it will affect intracellular pathways such as K-Ras [10–12].

Chronic inflammation triggers cellular events that can promote malignant transformation of cells and carcinogenesis. Epithelial mesenchymal transition (EMT) in human intestinal tumor progression is associated with the upregulation of the intermediate filament protein vimentin. Since vimentin is integral for the structural integrity of the cell [24, 25, 27] and adhesion signaling [8, 25], downregulating vimentin expression is sufficient to alter cell morphology [27, 28] as well as inhibiting cell motility and invasion [28]. This finding indicated that in colitis associated colon cancer undergoing EMT. There is growing evidence here that E-cadherin plays a crucial role in human cancer invasion and metastasis [24, 25, 27, 28]. The stimulation of E-cadherin while simultaneously attenuating expression of vimentin and MMP, suggests blocking of EMT and inhibiting the viability and motility.

One of the targeted therapies for colorectal cancer is cetuximab which works by inhibiting the growth of cancer cells by targeting EGFR, so that there is inhibition in the MAPK pathway and decreased K-Ras expression. In this study, cetuximab was combined with coelomic fluid from *Eisenia fetida*. Coelomic fluid has been known to be used as a therapy for several diseases because it has anti-inflammatory, antioxidant, antibacterial, and antitumor effects [7]. Biological substances contained coelomic fluid including lectin [8] and earthworm fibrinolytic enzyme also known as lumbrokinase [26, 29, 30]. Lectins have been proven to be used as anticancer in the MCF-7 cell line via the MAPK pathway by targeting EGFR to induce cell apoptosis [25]. A previous in vitro and in vivo study has shown that earthworm fibrinolytic enzyme component A from *Eisenia fetida* could effectively inhibit the proliferation of MCF-7 cells. Another study showed that earthworm fibrinolytic enzyme has significant antitumor activity by inhibiting the expression of matrix metalloproteinase-2 (MMP-2) [30]. MMP-2 (gelatinase A) and MMP-9 (gelatinase B) are both cancer associated. Gelatinase cleave many targets that regulate key signaling in cell growth, migration, invasion, inflammation, and angiogenesis [31]. So it is possible that there might be a change in MMP expression via activation of RUNX3 signaling [30]. Downregulation of *β*-catenin expression can cause a decrease in vimentin expression, and this is also in accordance with our results (Figures 2 and 5). Thus, the combination of cetuximab and CFEF can work synergistically.

K-Ras is also an effector molecule responsible for signal transduction from EGFR. EGFR plays an important role in controlling the proliferation of cancer cells and also metastasis [19]. An increase in K-Ras will increase proliferation and metastasis. This is consistent with the results that K-Ras increased significantly in DSS/AOM-induced mice. One of the things that is approved for cancer metastases is impaired activation of EMT. This shows that targeting EMT can be a promising therapeutic strategy for patients with colorectal cancer [20]. This is also consistent with the results shown by increasing of vimentin expression in DSS/AOM-induced mice. The improving of vimentin can occur through pathways involving *β*-catenin and TGF-*β*. *β*-Catenin increases EMT through activation of the vimentin signal pathway [25–28, 32]. Epithelial mesenchymal transformation during development and oncogenesis is an essential biological process. Downregulating E-cadherin and raising the mesenchymal marker vimentin are main transitional factors [24, 25, 27].

RUNX3 has been shown to inhibit EMT, which encourages metastasis; by regulating these signaling pathways, RUNX3 plays a critical role in the regulation of tumor cell migration, invasion, and proliferation from epithelial to mesenchymal [25, 28]. RUNX3 is ideally inactivated in lung adenocarcinoma induced by K-Ras, suggesting its potential role as a tumor suppressor in lung adenocarcinoma [29, 32]. In this study, we found that overexpression of RUNX3 significantly inhibited motility and invasiveness in CAC cells.

Our finding indicated that overexpression of RUNX3 could prevent EMT [27, 28, 32]. We examined EMT markers at the protein levels in the RUNX3-overexpressing cells. Cancers provide evidence that expression of RUNX3 in metastatic tissue has decreased significantly. This indicates that RUNX3 plays an important role in tumorigenesis and progression. The expression of protein RUNX3 was found to be substantially associated with decreased survival of CRC patients [25, 27, 28, 32]. RUNX3 overexpression is associated with decreased breast cancer cell invasiveness [26–29, 32]. The overexpressing RUNX3 cells had reduced invasive potential of the cells. RUNX3 is important in regulation of motility and invasiveness. Finally, functional experiments revealed that restoration of RUNX3 in CAC cells suppressed cell invasion and migration and regulated EMT-vimentin by CFEF.

RUNX3 overexpression is associated with decreased breast cancer cell invasiveness [26–29, 32]. The overexpressing RUNX3 cells had reduced invasive potential of the cells. RUNX3 is important in the regulation of motility and invasiveness. Finally, functional experiments revealed that restoration of RUNX3 in CAC cells suppressed cell invasion and migration and regulated EMT-vimentin by CFEF.

The present findings indicated that CFEF may serve as a novel agent for colon cancer treatment. The in vivo effects of anticancer as well as the potential therapeutic effectiveness of CFEF are worth further exploring. The earthworm crude extract is shown to have the ability to kill cancer cells directly in vitro [8, 9, 33] and to prevent the incidence and growth of tumor in vivo [10, 33, 34]. In addition, earthworm proteases have been shown to improve the therapy effects by both radiations therapy and chemotherapy.

Recently, a glycosylated component is separated from the earthworm *E. fetida* by Liu et al. [30], which has relations with apoptosis of tumor cells. It is identified to be a plasmin and also a plasminogen activator. The earthworm protease possesses obvious antitumor activity. It has been found that the earthworm protease can induce apoptosis of hepatoma cells and downregulate the expression of matrix of matrix metalloproteinase.

## 5. Conclusion

In conclusion, this study showed that the combination of the CFEF and cetuximab is able to inhibit proliferation by reducing K-Ras and vimentin expression in colorectal cancer in vivo. This finding maybe offers a promising method for a new anticancer therapy concept, especially for colorectal cancer treatment.

## Figures and Tables

**Figure 7:**
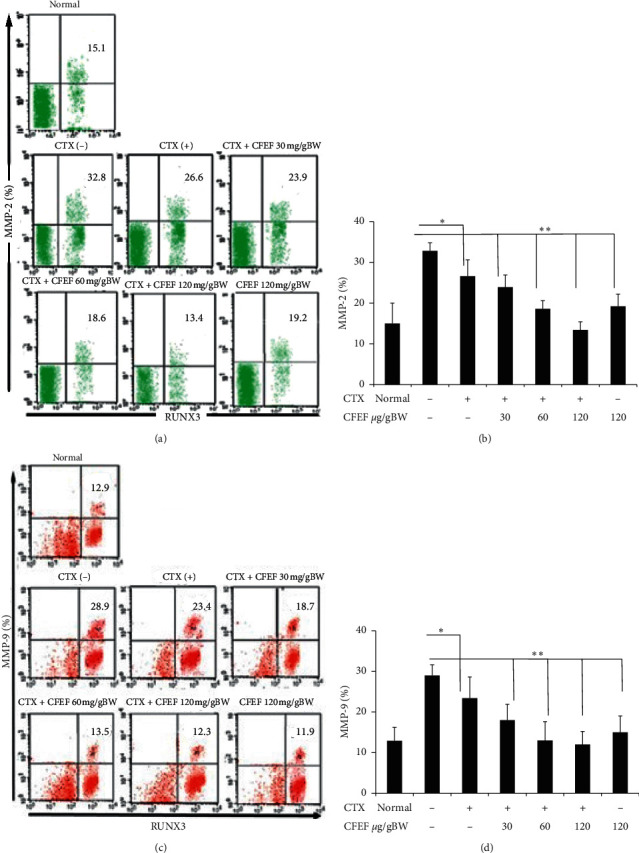
CFEF-cetuximab combination therapies were dependent RUNX3 to reduce MMP-2 and MMP-9 percentage in CECs cell. (a) Representative percentage of MMP2 after therapies with cetuximab and various concentrations of CFEF (30, 60, and 120 mg/g BW) in each histogram was shown and analyzed using flow cytometry (FACS Calibur, Becton Dickinson). Cells were gated from double positive cells (K-Ras^+^ and vimentin^+^ populations) and those populations that express RUNX3 were analyzed. Percentage of RUNX3^+^ MMP-2^+^ cell in each quadrant is shown inside the panels. (b) The percentage of RUNX3^+^ MMP-2^+^ cell reduced in CECs on combination therapy groups compared with cetuximab single group. (c) Representative percentage of RUNX3^+^ MMP-9^+^ cell reduced in CECs after combination therapy. Percentage of RUNX3^+^ MMP-9^+^ cell in each quadrant is shown inside the panels. (d) The percentage of RUNX3^+^ MMP-9^+^ cell decreased on combination therapy groups compared with cetuximab single group. Results shown are mean ± SD, with *n* = 6 replicates in each group. ^*∗*^*P* < 0.05, ^*∗∗*^*P* < 0.001.

## Data Availability

The data used to support the findings of this study are included within the article.

## Abbreviation

AOM: Azoxymethane
CAC: Colitis associated colon cancer
CFEF: Coelomic fluid *Eisenia fetida*
EGFR: Epidermal growth factor receptor
DSS: Dextran sulfate sodium
EMT: Epithelial mesenchymal transition
CECs: Intestinal epithelial cells
K-Ras: Kirsten-rat sarcoma viral oncogene homolog
MAPK: Mitogen-activated protein kinase
RUNX3: Runt-Related Transcription Factor-3.
